# Gas Sensing with Solar Cells: The Case of NH_3_ Detection through Nanocarbon/Silicon Hybrid Heterojunctions

**DOI:** 10.3390/nano10112303

**Published:** 2020-11-21

**Authors:** Giovanni Drera, Sonia Freddi, Tiziano Freddi, Andrea De Poli, Stefania Pagliara, Maurizio De Crescenzi, Paola Castrucci, Luigi Sangaletti

**Affiliations:** 1 I-Lamp and Dipartimento di Matematica e Fisica, Università Cattolica del Sacro Cuore, via dei Musei 41, 25121 Brescia, Italy; giovanni.drera@unicatt.it (G.D.); sonia.freddi@unicatt.it (S.F.); tizi_freddi@hotmail.it (T.F.); andrea.depoli@unicatt.it (A.D.P.); stefania.pagliara@unicatt.it (S.P.); 2 Department of Chemistry, Division of Molecular Imaging and Photonics, KU Leuven, Celestijnenlaan 200F, 3001 Leuven, Belgium; 3 Dipartimento di Fisica, Università di Roma Tor Vergata, 00133 Roma, Italy; decrescenzi@roma2.infn.it (M.D.C.); castrucci@roma2.infn.it (P.C.)

**Keywords:** carbon nanotubes, PV cells, CNT/Si heterojunctions, gas sensing, NH_3_

## Abstract

Photovoltaic (PV) cells based on single-walled carbon nanotube (SWCNT)/silicon (Si) and multiwalled carbon nanotube (MWCNT)/Si junctions were tested under exposure to NH_3_ in the 0–21 ppm concentration range. The PV cell parameters remarkably changed upon NH_3_ exposure, suggesting that these junctions, while being operated as PV cells, can react to changes in the environment, thereby acting as NH_3_ gas sensors. Indeed, by choosing the open-circuit voltage, V_OC_, parameter as read-out, it was found that these cells behaved as gas sensors, operating at room temperature with a response higher than chemiresistors developed on the same layers. The sensitivity was further increased when the whole current–voltage (I–V) curve was collected and the maximum power values were tracked upon NH_3_ exposure.

## 1. Introduction

Recent studies on photovoltaic devices have drawn attention to the possibility of combining photovoltaics with sensing properties with the aim of developing multifunctional devices. The capability of power generation is regarded as particularly attractive for diffuse monitoring grids, remote sensing, and portable devices [1,2,3,4,5].

In the rather heterogeneous set of devices proposed so far, hybrid carbon nanotube (CNT)/silicon (Si) heterostructures [6,7,8,9,10,11,12] have shown to be a promising platform to explore these concepts. Because of the sensitivity of electrical parameters to a flux of molecules over the CNT layer, it has also been suggested that these heterojunctions can be regarded as multifunctional devices, where the sensing capability can be combined with power generation of the junction [13,14,15,16,17,18,19,20] with the aim of obtaining self-powered devices [15,21,22] after proper device engineering.

Among possible target gas molecules, ammonia detection can address quite important issues in environmental monitoring [23,24,25,26,27,28,29], mostly in urban environment, as well as in breath analysis (i.e., breathomics), a noninvasive diagnostic method suitable for repeated measurements, which might provide a powerful tool for personalized medicine, including molecular phenotyping of respiratory diseases [30]. Continuous monitoring of ammonia atmospheric concentrations in urban areas is aimed at detecting usually low average concentrations (in the sub-ppm range) [25], mostly ascribed to vehicles emissions [26]. Furthermore, a growing body of investigation has highlighted the relevant role played by ammonia as a precursor of secondary fine particulate (PM10 and PM2.5) [27,28,29]. In the field of breathomics, a higher concentration of ammonia in human breath with respect to reference healthy patients can be related to liver or kidney disease [31,32,33,34,35], while a lower concentration of ammonia has been tracked in patients affected by chronic obstructive pulmonary disease [36].

**Figure 1:**
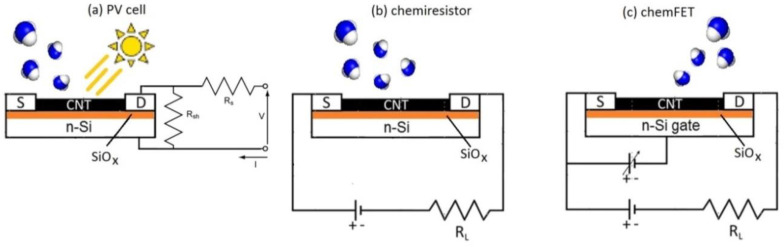
Layout of the read-out setup in a photovoltaic (PV) cell (**a**), chemiresistor (**b**) and chemical field-effect transistor (chem-FET) configurations (**c**). The n-doped silicon (Si) layer in (**c**) acts as the gate in the chem-FET, while it represents the n-side of the junction in (**a**). The p-side of the junction in (**a**) is represented by the carbon nanotube (CNT) layer. In all schemes, the SiO_x_ layer between Si and CNT is expressly shown. Contact pads (S and D) can be operated as source and drain in chem-FET (**c**), while they are used as metallic contacts in (**b**) to collect the current induced by gas exposure. In (**a**), these pads are equivalently used as contacts for the p-type layer of the junction. R_S_, R_sh_, and R_L_ are defined as the series, shunt, and load resistance, respectively.

CNTs are quite often used to detect ammonia target molecules, both as active layers in chemiresistors (CRs) and in chemical field-effect transistors (chem-FETs) [37,38,39,40,41,42,43]. Figure 1 shows a drawing of the read-out scheme for CNT layers in chemiresistor (mid panel) and chem-FET (right panel) configurations. Further sensitivity of these devices can be achieved by CNT functionalization with, e.g., oxide nanoparticles [42,43,44,45], thereby increasing the possible impact of these sensors in the field of ammonia detection.

The goal of the present study was to test the capability of the PV cell read-out scheme to provide quantitative tracking of exposure to NH_3_, presenting a proof-of-concept on the use of PV cells based on CNT/Si heterojunctions as gas sensors. With this purpose, PV cells based on single-walled CNT (SWCNT)/Si and multiwalled CNT (MWCNT)/Si junctions were tested under exposure to NH_3_ in the 0–21 ppm range. It was found that the PV cell parameters remarkably changed upon NH_3_ exposure at room temperature, suggesting that these junctions, while being operated as PV cells, can react to changes in the environment, thereby acting as ammonia gas sensors. In the case of the SWCNT/Si junction, response to NH_3_ was higher than that obtained from the same SWCNT layer by measuring the sheet resistance of this layer alone (CR read-out), indicating that, where applicable, this approach can be used to enhance sensitivity to ammonia of a CNT layer.

## 2. Materials and Methods

### 2.1. PV Cell Preparation

Chemical-vapor-deposited MWCNT powder (assay >90%, diameter: 5–9 nm) was purchased from Nanocyl, (Sambreville, Belgium) while highly pure polychiral SWCNT powder (assay >90%, diameter: 0.7–0.9 nm) was purchased from Sigma Aldrich. MWCNT and SWCNT powders were dispersed in an aqueous solution (30 μg mL^−1^) with 2% w/v sodium dodecyl sulfate (Sigma Aldrich, assay >98.5%) anionic surfactant. In order to disperse the suspension, both solutions were tip-ultrasonicated (Branson S250A, 200 W, 20% power, 20 KHz, Brookfield, CT, USA) in an ice bath for an hour, and a pipette was used to collect the unbundled supernatant. The result was a well-dispersed suspension, which was used to prepare a MWCNT film or a SWCNT film, as described in [46] and [47], respectively.

Using dry-transfer printing from a cellulose filter, the PV cells were prepared by depositing either a MWCNT film or a SWCNT film on hydrogen fluoride (HF)-etched bare Si window delimited by a SiO_2_(300 nm)/Cr(5 nm)/Au(150 nm) electrode. The dry-printing deposition technique consisted of soaking the CNT film with ethanol in order to improve its adhesion and then pressing it onto a substrate (HF-etched Si wafer in this case) with a glass slide. After few minutes, the dried cellulose filter was removed by peeling it off, thus leaving the CNT film adhered to the substrate.

The Si substrate was n-type (p∼3–12 Ω cm, N_D_∼6 × 10^14^ cm^−3^) with a Cr/Au ohmic back contact. The active area of the device was 0.09 cm^2^ for the SWCNT/Si heterojunction and 0.04 cm^2^ for the MWCNT/Si one. The MWCNT film on Si was 22.7 ± 0.4 nm thick, while the Si substrate was 54 μm thick. The power conversion efficiency (PCE) was 8 ± 1% without doping, while the PCE reached 10 ± 1% after exposure for 60 s to HNO_3_ vapors (additional data are reported in [46]). The SWCNT layer of 32 ± 5 nm thickness was deposited on a 114 μm thick silicon wafer, resulting in a PCE of 6 ± 1%, which was increased to 8 ± 1% after exposure for 15 s to HNO_3_ vapors. Once exposed to air, an oxidation of Si at the Si/CNT interface was observed through X-ray photoemission measurements in both samples, with an estimated thickness of 2.0 ± 0.2 nm.

### 2.2. Gas Exposure

NH_3_ diluted in synthetic air was fluxed at 50 sccm into a vacuum-tight chamber (volume = 2 dm^3^) to test the device in the 0–21 ppm concentration range. The device was also tested in open air. During exposure in air, the temperature was maintained at 22 ± 1 °C and the relative humidity (RH) at 25.6 ± 0.1%. During exposure in the vacuum-tight chamber RH was in the 8–10% range. The chamber was equipped with an optical window and connected to a mass flow controller system, providing a stable flux of the required gases. During the measurements in open air reference data were also collected with a Figaro (Osaka, Japan) TGS 2602 metal-oxide-based chemiresistor, hereafter denoted as MOX, where a metal-oxide film was deposited onto an alumina substrate of a sensing chip, with an integrated heater providing local heating (about 300 °C) of the metal oxide sensing element during gas exposure.

### 2.3. Electrical Measurements

The current–voltage (I–V) curves of the PV cells were measured just after cell preparation by an AM1.5 solar simulator operating at 100 mW/cm^2^ and with a spectral range peak of around 500 nm. In turn, the I–V curves measurements of the device inside the vacuum-tight chamber were performed by collecting current–voltage curves under illumination from a halogen lamp with a power density of 11.4 mW/cm^2^. Light from the source was focused through a lens and passed through a glass viewport to reach the cell mounted inside the chamber. The layout of the electrical measurements is shown in Figure 1a. Measurements in the chemiresistor configuration were carried out on the basis of the electrical scheme shown in Figure 1b. These measurements can be regarded as sheet resistance measurements of the CNT layers alone. All measurements were carried out at room temperature. All cells investigated as ammonia sensors displayed the same dependence of I–V curve on ammonia concentration but to different extents depending on the cell conditions. Here, we selected the best performing cells among a batch of four for the SWCNT/Si cell and a batch of two for the MWCNT/Si cell.

## 3. Results and Discussion

**Figure 2:**
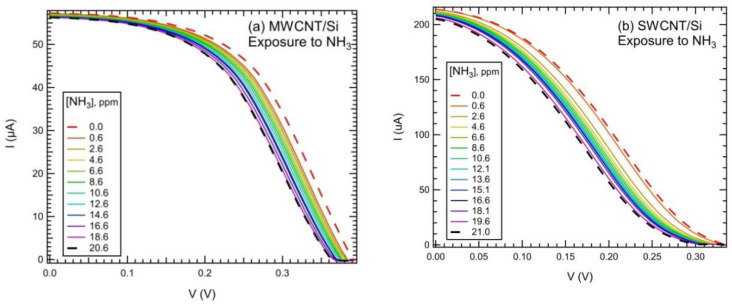
Effects of NH_3_ exposure (in the testing chamber) on the current–voltage (I–V) curves of multiwalled CNT (MWCNT)/Si (**a**) and single-walled CNT (SWCNT)/Si (**b**) hybrid PV cells. Maximum concentration in (**a**) was 20.6 ppm, while it was 21.0 ppm in (**b**). Intermediate concentration values are indicated in the inset of each panel.

Figure 2 shows changes in the I–V curves upon exposure to NH_3_ in the testing chamber for both the MWCNT/Si (Figure 2a) and SWCNT/Si (Figure 2b) cells. As can be observed, the curve line shape changes with gas concentration, along with short-circuit current (I_SC_) and open-circuit voltage (V_OC_). Exposure to NH_3_ resulted in an overall decrease in cell efficiency (mainly related to the decrease in I_SC_ and V_OC_ with ammonia concentration), but the major result was the sensitivity of these parameters to the interaction of the cell with NH_3_. Both experimental and theoretical studies recognized a p-type behavior of the SWCNTs when exposed to ambient atmosphere. This phenomenon is ascribed to oxygen molecule (O_2_) adsorption, which naturally p-dopes the nanotube [48,49,50,51]. The reduction of I_SC_ and V_OC_ was consistent with the p-doping of CNTs and with the reducing character of ammonia. The reverse behavior is expected for exposure to oxidizing NO_2_ molecules, as reported for similar cells in [52].

**Figure 3:**
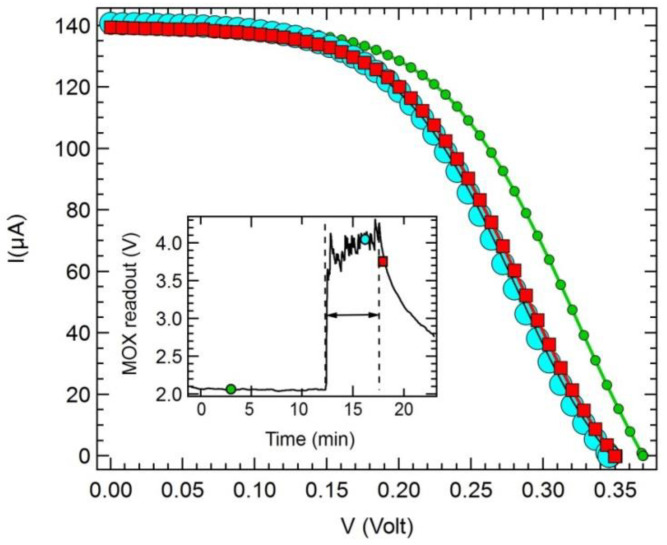
Example of I–V curves collected during different phases of SWCNT/Si PV cell exposure to ammonia in air. Green dots: before exposure, light blue spheres: maximum NH_3_ concentration, red squares: early recovery phase. In the inset, these phases are indicated on top of the response curve of the reference metal-oxide-based chemiresistor (MOX) gas sensor collected during exposure. The horizontal arrow denotes the exposure time (approximately 5 min).

These changes can be used to track exposure to gas and therefore to use the heterojunction as gas sensors. Indeed, Figure 3 details the I–V curves collected from the SWCNT cell at selected points of the exposure process in open air, i.e., before exposure, at the maximum concentration, and at the very beginning of recovery after exposure was stopped. The inset shows the exposure time window, along with the signal of the MOX reference sensor.

**Figure 4:**
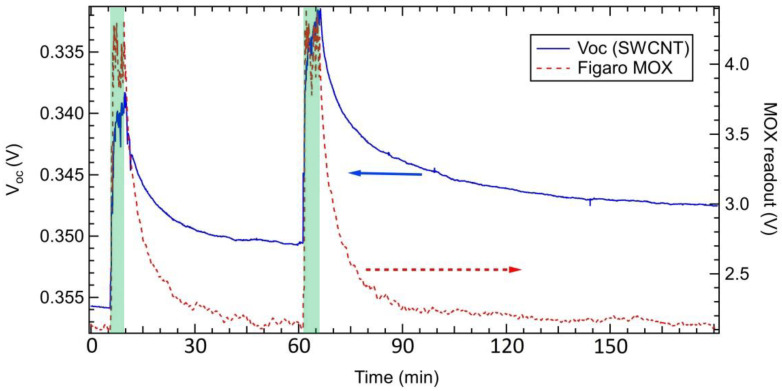
Sequence of exposures to NH_3_ in air tracked by the V_OC_ read-out on the SWCNT/Si PV cell (left axis, thick line) compared to the read-out of the Figaro MOX (right axis, dashed line). Data from the SWCNT PV cell were collected under white light illumination. The left axis has been reversed to allow for comparison with data from the Figaro MOX. The shaded areas represent the exposure phase.

Based on these results, a sequence of exposure to NH_3_ was carried out in air in order to explore the cell behavior in terms of rise time and recovery time, with data from the MOX sensor also collected as reference. As V_OC_ quantity displayed a major change, larger than the change detected for I_SC_, the PV cell response was tracked by collecting V_OC_ during exposure. Figure 4 provides an example of exposure to NH_3_ in air. Similar results were observed for MWCNT-based cells.

As discussed in detail in [52], the V_OC_ reduction can be ascribed to a decrease in the CNT work function due to electron transfer from NH_3_ to the CNT layer. According to the theory for a Schottky junction solar cell, the V_OC_ can be related to the barrier height Φ_B_ at the interface as follows:

V_OC_ = n Φ_B_ + (nkT/q)ln(J_L_/A* T^2^) (1)

where n is the junction ideality factor, T is the temperature, k is the Boltzmann constant, J_L_ is the junction saturation current density, and A* is the Richardson constant. The barrier height Φ_B_ can be estimated from the difference between the work function of the CNT film and that of the silicon. Thus, the change in the work function of the nanotube through adsorption is expected to influence the cell performance through a change of the barrier height. In the case of ammonia absorption, theoretical calculations [52] have shown that NH_3_ molecules down-shift the CNT work function, ultimately leading to a V_OC_ reduction.

With regard to signal recovery after exposure, while a full recovery of the read-out value before exposure was reached in about 1 h for the MOX sensor, full recovery was not observed within 1 h for the CNT-based sensor, although V_OC_ reached a constant value. We ascribe this difference to the different operating temperatures of the sensors. While the sensing layer of the MOX sensor was heated to induce full desorption of the adsorbed target molecules, the CNT-based sensor was operated at room temperature, so the stable behavior observed after about 1 h can be ascribed to spontaneous desorption at RT.

**Figure 5:**
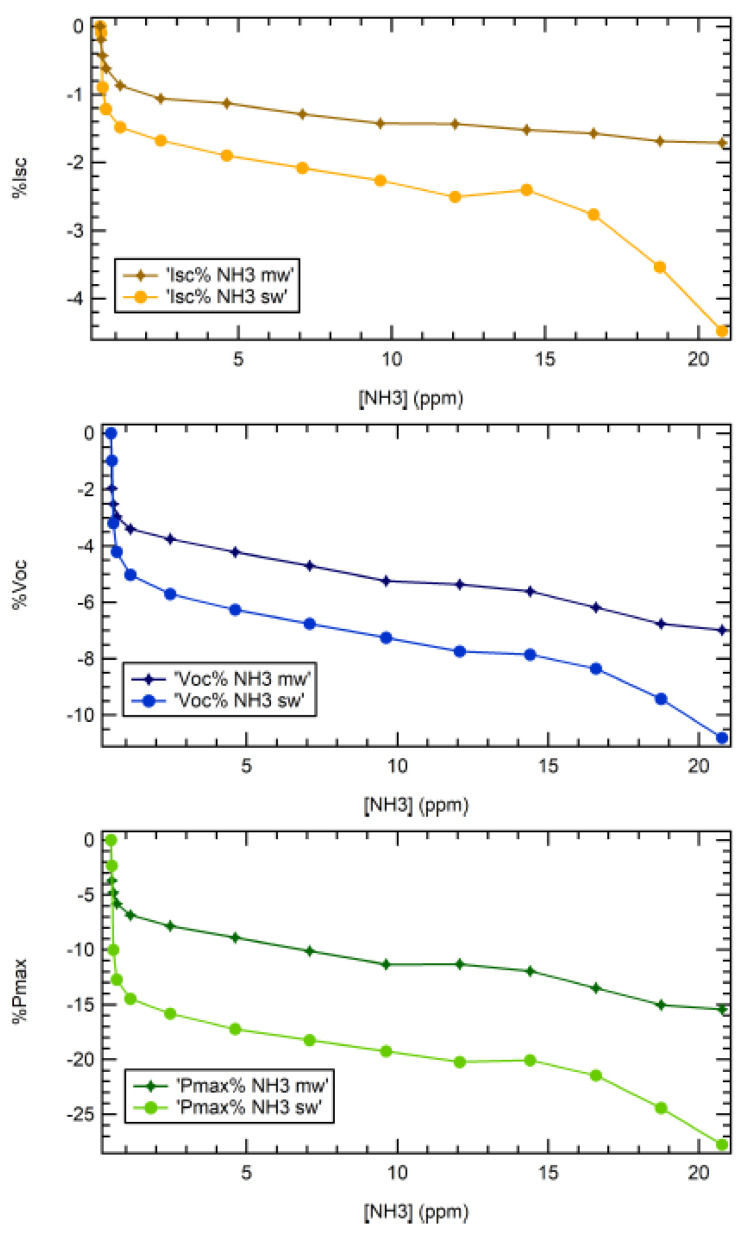
I_sc_, V_oc_, and P_max_ dependence on NH_3_ concentration (0–21 ppm range) for MWCNT (diamonds) and SWCNT (dots) PV cells. Values are expressed as a percentage of the parameter (I_sc_, V_oc_, and P_max_) change with respect to the read-out before exposure. All data were collected in the testing chamber.

Following these measurements, both cells were then systematically investigated upon exposure to NH_3_ in the testing chamber in order to determine the sensor calibration curve in the 0–21 ppm range. The results are shown in Figure 5, where the relative variation of I_SC_, V_OC_, and P_max_ is tracked vs. NH_3_ concentration. The lowest concentration measured was 0.6 ppm for both devices. The point at 0 ppm corresponded to the read-out before exposure. Fluctuations due to electronic read-out were below 0.1% of the value measured before exposure. As can be observed from the data presented in Figure 5, the biggest change was displayed by the P_max_ curve (28% for SWCNT and 15% for MWCNT) of the initial value, while the smallest change was displayed by the I_sc_ value (4.5% and 1.8% for SWCNT and MWCNT, respectively). Here, I_sc_ and V_oc_ could be monitored separately in real time during exposure, while P_max_ was extracted from each I–V curve by identifying the maximum of the I–V product vs. the V curve.

All responses to ammonia, registered in terms of I_SC_, V_OC_, and P_max_ changes, presented a nonlinear behavior, which is quite common in the field of gas sensing with CNTs. In the low-concentration limit, this behavior is usually fitted by a Freundlich curve [53,54,55], a power law curve where, for a CR read-out, ΔR/R_0_ = A[NH_3_]^p^, with A being a normalization constant and [NH_3_] being the target gas concentration. The *p* value is usually found in the 0 < *p* < 1 range (i.e., sublinear behavior).

**Figure 6:**
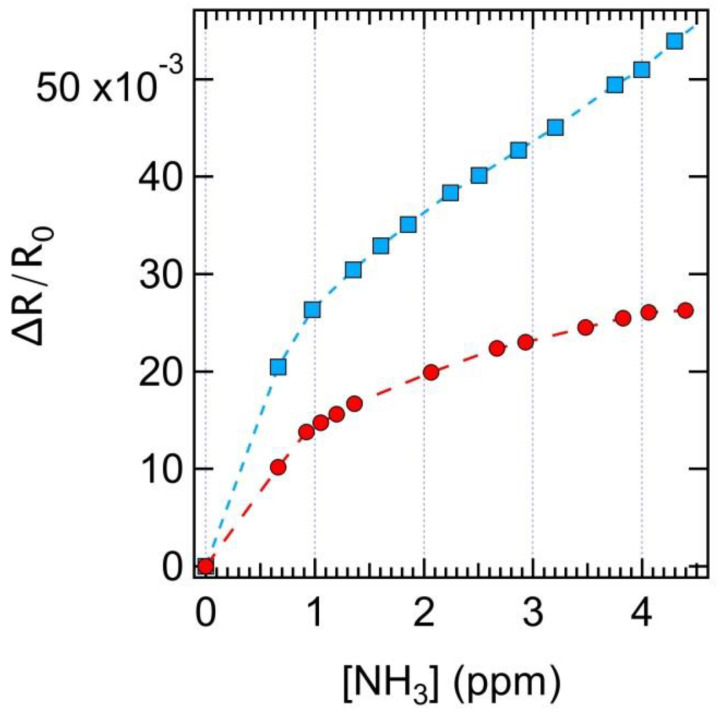
Response ΔR/R_0_ of the SWCNT layer as chemiresistor in dark conditions (red circles) and under white light illumination (blue squares). NH_3_ exposure in the 0.6–4.5 ppm range. Dashed lines are drawn as a guide for the eye. All data were collected in the testing chamber.

At this stage, it is rather important to compare the performance of the sensors in the PV cell read-out with respect to the chemiresistor read-out. Figure 6 shows the experimental data collected in the testing chamber from the SWCNT cell in the chemiresistor configuration read-out. The two curves were collected in both dark and light conditions during exposure to ammonia in the 0–4.7 ppm range.

While ΔR/R changes were observed in both cases, the largest variation in the chemiresistance was observed under illumination, as reported for selected concentration values in Table 1. In any case, these values were well below those observed for the ΔP_max_ at the corresponding concentration values, showing that operation as a PV cell provided sensitivity about three times larger than the CR read-out under the same illumination conditions. The difference between the behavior observed in dark and ambient light conditions can be ascribed to the presence of the heterojunction effect, which, in spite of not being directly involved in CR measurements, can provide charge injection (namely holes from Si to the CNT side [56]) whenever photons are absorbed at the heterojunction during CR measurements under light conditions. When this occurs, holes migrate to the p-side of the junction (i.e., the CNT side), increasing the probability of electron transfer from NH_3_ to CNT, thereby enhancing the resistance increase in the CNT layer. Therefore, the illuminated junction affects the ammonia detection both when the device is operating as a PV cell and as a chemiresistor. The larger effect observed in the former case can be related to a reduction of R_sh_, the parallel resistance in the equivalent circuit of the cell (Figure 1a), yielding a voltage drop across the junction. Equivalently, the effect of interaction with ammonia on charge transport across the junction can be rationalized in terms of a change in the CNT work function [52].

When properly normalized to NH_3_ concentration (1.0 ppm or 4.5 ppm), our device performed quite well compared to the many CNT-based sensors reviewed in [40]. A benchmark of the current CNT/Si junction sensor with respect to CNT-based chemiresistor performance is shown in the Supplementary Materials (SM) as Figure S1. Data benchmarking was carried out by considering the sensitivity, S, defined as S = 100 × (ΔR/R_0_)/ [NH_3_], where S values are reported as %/ppm.

The present device could therefore provide five channels to track the ammonia concentration: (i) sheet resistance in dark conditions, (ii) sheet resistance under illumination, (iii) V_OC_, (iv) I_SC_, and (v) P_max_ under illumination. The first two were part of the CR readout scheme, while the remaining three were part of the PV cell readout scheme.

Limitations of the present device can be identified in the recovery value after exposure to NH_3_. As shown in Figure 4, although the recovery reached a stable value in a time comparable to that observed for the MOX sensor, this value was higher than the R_0_ measured just before each exposure. We ascribe this effect to the operation at room temperature, while the MOX was operated with a heater that enabled full recovery after exposure had stopped. This point deserves further investigations, mostly in light of possible detrimental effects on the cell performance at high temperatures. Indeed, an increase of temperature is known to negatively affect PV cell performance [57]. A further aspect to investigate in view of the use as a gas sensor is the cell stability. This kind of PV cells are usually tested for a few weeks but without exposure to specific polluting gases. The best performing cells show remarkable stability over this time (see, e.g., figure 13 in [8]), in spite of having the junction exposed to some extent to ambient air that can reach the Si/CNT interface by diffusing through the CNT bundle layer. At the moment, the long-term stability of this interface after repeated exposure to target gas molecules has not yet been assessed. Finally, our read-out scheme allowed us to collect changes in V_OC_ and I_SC_ during exposure to NH_3_ with a rate of 1 Hz. Examples of these V_OC_ read-outs are provided in Figure 3 and Figure 4. As for P_max_, we needed to collect the whole I–V curve and then extract P_max_ from the IV–V (i.e., P–V) curve. We could register an I–V curve in 25 s, which is compatible for breathomics and environmental sensing applications addressed in the Introduction. However, this might not be fast enough for alarm sensors, where a quicker response could be required to cope with safety standards.

## 4. Conclusions

With respect to previous works [15,16,17,18,19], in the present paper, we focused on the possibility of using PV cells based on SWCNT/Si and MWCNT/Si as an ammonia gas sensor, taking advantage of the PV cell read-out scheme to measure changes in the PV cell electrical properties during exposure to the target gas.

It was found that the PV cell parameters measured at room temperature showed remarkable changes upon NH_3_ exposure in the 0–21 ppm range. The use of this scheme is novel compared to those (CR and FET) currently used and reported in the literature. Further measurements were specifically carried out to determine to what extent this device could be used as a gas sensor. In particular, we presented the response vs. time behavior and the calibration curves. In this regard, our investigation can be regarded as a proof-of-concept of the possible use of CNT/Si-based PV cells as gas sensors. In the case of the SWCNT-based device, we compared the results obtained in the PV cell read-out scheme with those obtained on the same device with a conventional CR read-out scheme, showing the better performance obtained in the former case. Finally, benchmarking with respect to other CNT-based CR sensors showed remarkable performances in terms of sensitivity at two reference NH_3_ concentrations (1.0 and 4.5 ppm). Therefore, where applicable, this approach can be used to enhance the sensitivity to ammonia of a SWCNT layer.

## Figures and Tables

**Table 1:** Response of the SWCNT layer in the chemiresistive (CR_D_ (dark conditions) and CR_L_ (under ambient light)) and PV read-out configurations.

[NH_3_]	CR_D_ (ΔR/R_0_, %)	CR_L_ (ΔR/R_0_, %)	PV (ΔP_max_/P, %)
1.0 ppm	1.5	2.6	10
4.5 ppm	2.6	5.6	17

## Supplementary Materials

The following are available online at https://www.mdpi.com/2079-4991/10/11/2303/s1, Figure S1: Benchmarking of the sensor performances towards NH_3_ detection.
