# Supplementary material for: Gas Sensing with Solar Cells: The Case of NH3 Detection through Nanocarbon/Silicon Hybrid Heterojunctions
Source: Nanomaterials (Basel). 2020 Nov 21;10(11):2303. doi: 10.3390/nano10112303 (PMC7700682; doi:10.3390/nano10112303)
Supplement: Supplementary file 1 [file nanomaterials-10-02303-s001.pdf]

nanomaterials

Supplementary Materials

Gas Sensing with Solar Cells: The Case of NH_3 Detection through Nanocarbon/Silicon Hybrid Heterojunctions

Giovanni Drera [1], Sonia Freddi [1,2], Tiziano Freddi [1], Andrea De Poli [1], Stefania Pagliara [1], Maurizio De Crescenzi [3], Paola Castrucci [3] and Luigi Sangaletti [1,*]

[1] I-Lamp and Dipartimento di Matematica e Fisica, Università Cattolica del Sacro Cuore, via dei Musei 41, 25121 Brescia, Italy; giovanni.drera@unicatt.it (G.D.); sonia.freddi@unicatt.it (S.F.); tizi_freddi@hotmail.it (T.F.); andrea.depoli@unicatt.it (A.D.P.); stefania.pagliara@unicatt.it (S.P.)

[2] Department of Chemistry, Division of Molecular Imaging and Photonics, KU Leuven, Celestijnenlaan 200F, 3001 Leuven, Belgium

[3] Dipartimento di Fisica, Università di Roma Tor Vergata, 00133 Roma, Italy; decrescenzi@roma2.infn.it (M.D.C.); castrucci@roma2.infn.it (P.C.)

* Correspondence: luigi.sangaletti@unicatt.it

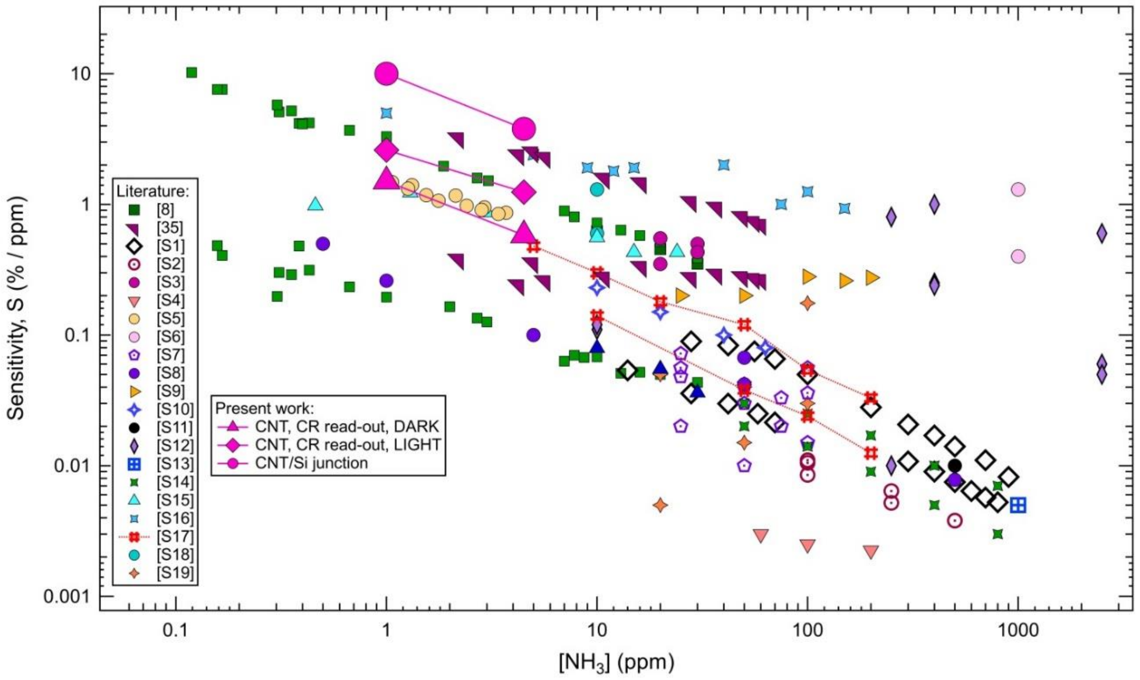

Figure S1. Benchmarking of the current CNT/Si junction sensor with respect to CNT-based chemiresistor performances (adapted from Figure 8, Nanotechnology **28** (2017) 255502 © IOP Publishing. Reproduced with permission. All rights reserved). Purple circles: CNT/Si junction; purple rhombus: chemiresistor (CR) readout under illumination; purple triangles: chemiresistor (CR) readout in dark conditions; Refs [8] and [35] refer to the main paper bibliography.

References

S1. Nguyen LQ, Phan PQ, Duong HN, Nguyen CD and Nguyen LH, 2013 Sensors 13 1754.

S2. Majzlíková P et al., 2015 Sensors 15 2644.

S3. Sharma S, Hussain S, Singh S and Islam SS, 2014 Sensors Actuators B 194 213.

S4. Chen D-J, Lei S, Wang R-H, Pan M and Chen Y-Q 2012 Chin, J. Anal. Chem. 40 145.

S5. Rigoni F, Tognolini S, Borghetti P, Drera G, Pagliara S, Goldoni A and Sangaletti L, 2013 Analyst 138 7392.

S6. Lee H, Lee S, Kim DH, Perello D, Park Y J, Hong SH, Yun M and Kim S, 2012 Sensor 12 2582.

S7. Han JW, Kim B, Li J and Meyyappan M, 2014 RSC Adv. 4 549.

S8. Randeniya LK, Martin PJ, Bendavid A and McDonnell J, 2011 Carbon 49 5265.

S9. Jung HY, Jung SM, Kim JR and Suh JS, 2007 Appl. Phys. Lett. 90 153114.

S10. Guerin H, Poche HL, Pohle R, Bernard LS, Buitrago E, Ramos R, Dijon J and Ionescu AM, 2014 Carbon 78 326.

S11. Valentini L, Cantalini C, Armentano I, Kenny JM, Lozzi L and Santucci S, 2004 Diam. Relat. Mater. 13 1301.

S12. Liu Z, Liao G, Li S, Pan Y, Wang X, Weng Y, Zhang X and Yang Z, 2013 J. Mater. Chem. A 1 13321.

S13. Ling Y, Zhang H, Gu G, Lu X, Kayastha V, Jones CS, Shih WS and Janzen DC, 2014 IEEE Sens. J. 14 1193.

S14. Lin ZD, Young SJ and Chang SJ, 2016 IEEE Trans. Electron. Devices 63 476.

S15. Rigoni F, Drera G, Pagliara S, Goldoni A and Sangaletti L, 2014 Carbon 80 356.

S16. He L, Ji Y, Meng F, Li M and Liu J, 2009 Mater. Sci. Eng. B 163 76.

S17. Battie Y, Ducloux O, Thobois P, Dorval N, Lauret J S, Attal-Tretout B and Loiseau A, 2011 Carbon 49 3544.

S18. Dong KY, Choi J, Lee YD, Kang BH, Yu YY, Choi HH and Ju BK, 2013 Nanoscale Res. Lett. 8 12.

S19. Han JW, Kim B, Li J and Meyyappan M, 2013 Appl. Phys. Lett. 102 193104.
